# miR-21 and miR-145 as Prognostic Biomarkers for Radiotherapy Responses in Cervical Cancer Patients: A Preliminary Study

**DOI:** 10.3390/ijms251910545

**Published:** 2024-09-30

**Authors:** Andi D. Putra, Andrijono, Hariyono Winarto, Ani R. Prijanti, Lisnawati Rachmadi, Trevino A. Pakasi, Supriadi Gandamihardja, Jourdan Wirasugianto, Amelia

**Affiliations:** 1 Doctoral Program in Medical Sciences, Faculty of Medicine, Universitas Indonesia, Central Jakarta 10430, Indonesia; 2 Division of Gynecologic Oncology, Department of Obstetrics and Gynecology, Faculty of Medicine, Universitas Indonesia, Cipto Mangunkusumo Hospital, Central Jakarta 10430, Indonesia; andrijono@gmail.com (A.); hariyono.winarto@ui.ac.id (H.W.); 3 Department of Biochemistry and Molecular Biology, Faculty of Medicine, Universitas Indonesia, Cipto Mangunkusumo Hospital, Central Jakarta 10430, Indonesia; ani.retno@ui.ac.id; 4 Department of Anatomical Pathology, Faculty of Medicine, Universitas Indonesia, Cipto Mangunkusumo Hospital, Central Jakarta 10430, Indonesia; lisnawatidrsppa@gmail.com; 5 Department of Community Medicine, Faculty of Medicine, Universitas Indonesia, Central Jakarta 10310, Indonesia; trevino.ap@ui.ac.id; 6 Division of Gynecologic Oncology, Department of Obstetrics and Gynecology, Faculty of Medicine, Universitas Padjadjaran, Hasan Sadikin Hospital, Bandung 40161, Indonesia; supriadigand@yahoo.co.id; 7 Assistant Division of Gynecologic Oncology, Department of Obstetrics and Gynecology, Cipto Mangunkusumo Hospital, Central Jakarta 10430, Indonesia; jourdanwirasugianto@gmail.com (J.W.); amelsumber@gmail.com (A.)

**Keywords:** biomarker, cervical cancer, microRNA-21, microRNA-145, radioresistance

## Abstract

Radioresistance poses a significant challenge in the effective treatment of cervical cancer, often leading to poor patient outcomes. MicroRNA-21 (miR-21) and MicroRNA-145 (miR-145) are oncogenic micro-RNAs associated with various cancers, including cervical cancer, but their potential as predictive biomarkers for radioresistance remains underexplored. This study aimed to investigate the association between miR-21 and miR-145 expressions and the response to radiation therapy in cervical cancer patients. An analytical cross-sectional study was conducted on 140 subjects with cervical cancer stages IIIB and IVA who received definitive radiotherapy. miR-21 and miR-145 expressions were measured using real-time reverse transcriptase–polymerase chain reaction (RT-qPCR). A total of 102 subjects (72.9%) were classified as having stage III cervical cancer, and 38 subjects (27.1%) were classified as having stage IV cervical cancer. Disease progression occurred in 60.7% of subjects. The cut-off value for miR-21 expression was 0.00088 nmol/(mg/mL) (AUC 0.676, sensitivity 70.8%, specificity 50.8%), and a higher expression was significantly associated with radioresistance (*p* = 0.010). miR-145, with a cut-off of 0.0239 nmol/(mg/mL) (AUC 0.612, sensitivity 67.5%, specificity 45.5%), showed no significant association with treatment response (*p* = 0.132). Combining miR-21 and miR-145 (AUC 0.639, sensitivity 68.6%, specificity 46.9%, *p* = 0.063) did not significantly improve the predictive accuracy. This study suggests that an elevated miR-21 expression is significantly associated with radioresistance in cervical cancer patients, while miR-145 expression shows no significant correlation with treatment response. Additionally, combining miR-21 and miR-145 does not enhance the predictive power.

## 1. Introduction

Cervical cancer remains one of the most significant threats to women’s health globally, ranking as the eighth most commonly diagnosed cancer worldwide in 2022, with an estimated 662,301 cases and 348,874 deaths [1]. In 2022, cervical cancer ranked second in incidence in Indonesia, with 36,964 cases reported. The predominant histological subtypes are squamous cell carcinoma (SCC) and adenocarcinoma (AC), with SCC being substantially more prevalent [2]. Despite advancements in surgery, radiation, and chemotherapy, some cervical cancer patients still experience early metastasis, leading to a poor prognosis [3,4]. In Indonesia, most patients are diagnosed at stage IIIB [5], making radiotherapy the standard treatment. Although radiotherapy significantly improves outcomes, radioresistance remains a major cause of treatment failure [6]. Micro-RNAs (miRs) have been widely investigated in cancer due to their critical roles in tumor progression and response to radiotherapy [7].

miRs are small non-coding RNA molecules that regulate the gene expression post-transcriptionally by binding to target mRNAs, thus suppressing or enhancing their translation. miRs are natural riboregulators that can control the gene expression through the RNA interference (RNAi) pathway. This oncomiR, composed of 18–25 nucleotides, plays a critical role in cancer biology [8,9]. Ongoing studies show that miR affects pathogenesis, predictive variables, and the treatment of many malignancies, including gynecologic tumors [10,11,12]. Among 540 samples of human solid tumors involving cervical, lung, breast, stomach, prostate, colon, and pancreatic cancers, miR-21 stands out as the sole miR exhibiting elevated expression [13,14].

In cervical cancer, miR-21 acts as an oncomiR, initiating cellular proliferation and tumorigenesis [15,16]. miR-21 is located on chromosome 17q23.2, and its oncogenic role in cervical cancer is to bind to a target mRNA or long non-coding RNAs (lncRNAs). Moreover, signaling pathways in cervical cancer affected by miR-21 include tumor necrosis factor alpha (TNF-α)/caspase-3/caspase-8, phosphatidylinositol 3-kinase (PI3K)/protein kinase B (*Akt*)/mammalian target of rapamycin (mTOR), and rat sarcoma virus (RAS)/mitogen-activated protein kinase (MEK)/extracellular signal-regulated kinase (ERK) [17].

miR-145 is a tumor suppressor that targets various tumor-specific genes and proteins, affecting related signaling pathways. miR-145 is one of the miRs that are found to be deregulated in most female cancers [18]. The miR-145 gene resides on chromosome 5p32, and its expression is regulated by p53. The expression of miR-145 is often found to be downregulated in various tumor types, especially in cases with mutant p53. miR-145 is involved in the regulation of tumor growth, invasion, metastasis, angiogenesis, and tumor stem cell proliferation. The expression of miR-145 in various tumors is significantly lower than in normal tissues, and the overexpression of miR-145 inhibits the growth of various types of tumor cells, decreases tumor spreading ability, and improves tumor sensitivity to chemotherapeutic drugs [19,20].

While miR-21 and miR-145 have been studied in various cancers, their specific roles in radioresistant cervical cancer remain insufficiently understood. This study aims to fill this gap by exploring the prognostic potential of these miRs in patients who exhibit resistance to radiotherapy. Moreover, these findings may provide a basis for research on other cancers and allow for a comparison with existing studies.

## 2. Results

### 2.1. Clinical Data

A concise overview of the demographic characteristics of the patients is shown in Table 1. This research included a total of 140 subjects. The median age of the subjects was 52 years; 107 subjects (76.4%) were married, and 78 subjects (55.7%) had a parity greater than 2.

### 2.2. Correlation between Histopathology and Disease Staging towards Radiation Response

Among the 140 subjects, 102 (72.9%) were classified as having stage III cervical cancer and 38 (27.1%) as having stage IV cervical cancer. Subjects who were unresponsive to radiation were classified as having a progressive or stable disease, while those who responded were categorized into complete or partial response groups, according to the RECIST 1.1 criteria. Of the total cohort, 30.7% achieved a complete response, 8.6% had a partial response, and 60.7% experienced disease progression. Significant differences were observed in the proportions of the disease staging and the responses to radiation therapy among subjects with advanced cervical cancer, with *p* < 0.001. These variations underscore the importance of the disease stage in predicting treatment outcomes. However, the specific histopathological subtype of cervical cancer did not significantly impact the response to radiation therapy, with *p* = 0.617 (Table 2).

### 2.3. Relationship between miR-21 and miR-145 Level of Expression and Radiation Therapy Response

The expression of miR-21 and miR-145 was detected in 137 samples, while 3 samples did not show any detectable miR expression. The results revealed a significant increase in the expression of both miR-21 and miR-145 among subjects who did not respond to radiation treatment. The Mann–Whitney test confirmed that the radioresistant group had higher median expression levels and interquartile ranges for both miR-21 and miR-145 compared to the radiosensitive group, indicating greater variability within the radioresistant cohort. miR-21 was significantly elevated in the radioresistant group (*p* < 0.001), while miR-145 also showed a notable increase (*p* = 0.029), suggesting that both play a role in radiation resistance. In stage IIIB, miR-21 was elevated in the radioresistant group (*p* = 0.011), and in stage IVA, the increase was even more significant (*p* = 0.004). For miR-145, higher levels were observed in the radioresistance group for stage IIIB (*p* = 0.081) and stage IVA (*p* = 0.407), although these values did not reach statistical significance (Table 3).

The established cut-off values for miR-21 and miR-145 were 0.00088 and 0.0239 nmol/(mg/mL), respectively. Our Receiver Operating Characteristic (ROC) analysis revealed AUC values of 0.676 for miR-21 and 0.612 for miR-145, both indicating moderate predictive strength as biomarkers for radioresistance in advanced cervical cancer. miR-21 demonstrated a sensitivity of 70.8% and a specificity of 50.8%, while miR-145 exhibited a sensitivity of 67.5% and a specificity of 45.5%. The proportion of subjects with miR-21 levels above the cut-off value was significantly higher in the radioresistant group than in the radiosensitive group (*p* = 0.010), indicating that the miR-21 cut-off effectively distinguishes between the two groups in terms of radioresistance. Conversely, miR-145, with a *p*-value of 0.132, did not significantly differentiate between the groups, suggesting that its cut-off is less effective in predicting radioresistance outcomes.

**Figure 1:**
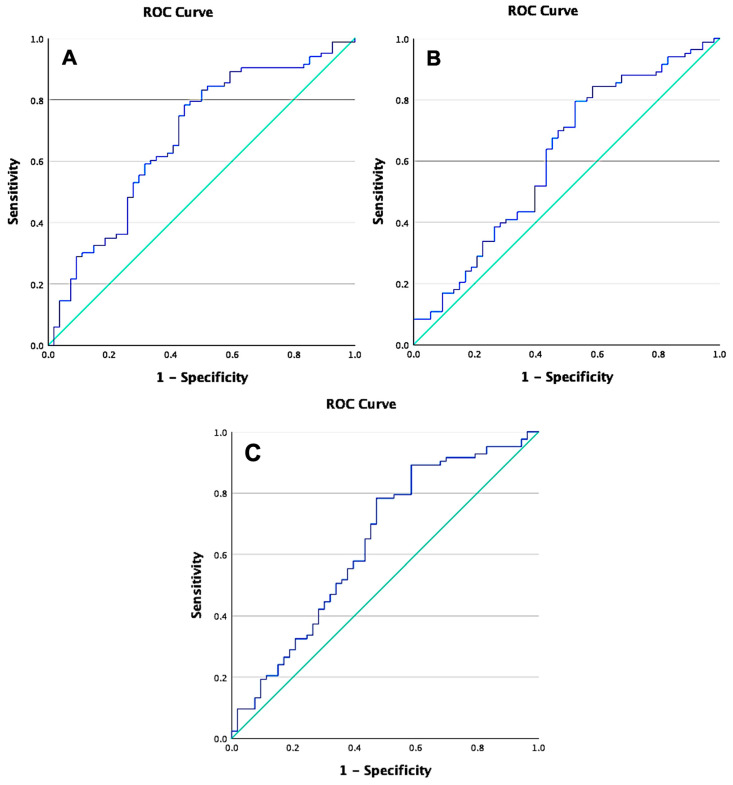
Receiver Operating Characteristic (ROC) curve of micro-RNAs. The blue line represents the true positive rate (sensitivity) against the false positive rate (1-specificity), while the green line indicates the reference line. (**A**) ROC curve for miR-21 shows an AUC of 0.676; (**B**) ROC curve for miR-145 shows an AUC of 0.612; (**C**) ROC curve for the combination of miR-21 and miR-145 shows an AUC of 0.639.

The combination of miR-21 and miR-145 yielded an AUC of 0.639 with a *p*-value of 0.063, suggesting that the predictive value did not reach statistical significance in distinguishing radioresistance. The sensitivity and specificity were 68.6% and 46.9%, respectively, indicating moderate predictive capabilities. However, the lack of statistical significance implies that these biomarkers together do not effectively differentiate between radioresistant and radiosensitive groups in advanced cervical cancer. These findings suggest that elevated miR-21 expression above the cut-off value could serve as a more reliable marker for predicting radioresistance in advanced cervical cancer (Figure 1).

## 3. Discussion

In this study, approximately 60.7% of individuals exhibited progressive disease according to the RECIST 1.1 criteria. The threshold for miR-21 was 0.00088 nm/(mg/mL) with an AUC of 0.676, and a significant difference in concentrations above the cut-off was found between the radioresistant and radiosensitive groups (*p* = 0.010). miR-21 demonstrated a sensitivity of 70.8%, indicating its relative effectiveness in identifying radioresistant patients. However, with a specificity of 50.8%, it shows only a moderate ability to exclude radiosensitive patients correctly. In contrast, miR-145 had a threshold of 0.0239 nm/(mg/mL) and an AUC of 0.612, with no significant difference between groups (*p* = 0.132). Its sensitivity was 67.5%, but the lower specificity of 45.5% resulted in higher misclassification rates. The combination of miR-21 and miR-145 yielded an AUC of 0.639 (*p* = 0.063), with a combined sensitivity and specificity of 68.6% and 46.9%, respectively. These findings suggest that while miR-21 may be more useful in predicting radioresistance than miR-145, their combination does not significantly enhance the predictive accuracy.

miR-21, classified as an oncomiR, plays a critical role in the carcinogenic process, causing upregulation in various cancers, including cervical cancer. The genes that are targeted include tropomyosin 1, phosphatase and tensin homolog (PTEN), and programmed cell death 4 (PDCD4). These miR-21 target genes are involved in malignancy traits such as cell invasion, metastasis, proliferation, and anti-apoptosis [21,22,23]. In cervical cancer, elevated miR-21 levels lead to decreased PTEN regulation, ultimately enhancing the growth and metastasis of tumor cells [24]. The dysregulation of miR-21 occurs during cervical cancer progression [15]. The high miR-21 concentrations in this study reflect patients with highly proliferative tumor cells that are prone to metastasis.

Additionally, miR-21 has been implicated in radioresistance through its inhibition of PTEN and PDCD4. Reduced PTEN expression, a target of miR-21, is known to cause radioresistance in lung cancer by encouraging tumor cell proliferation and invasion [25]. Similarly, miR-21 overexpression leads to decreased PDCD4 expression, contributing to radioresistance in lung cancer [26]. Resistances to platinum-based chemotherapy in patients with non-small cell lung cancer stages I-III are also associated with increased miR-21. Non-responsive patients exhibit higher miR-21 concentrations compared to responsive ones [27]. Aguilar-Martínez SY et al. reported that miR-21 plays a significant role in promoting cervical cancer cell proliferation and migration by downregulating the tumor suppressor gene reversion-inducing cysteine-rich protein with kazal motifs (RECK). Silencing miR-21 leads to increased RECK expression, reduced cell proliferation, and inhibited migration [28]. Another study by Liu et al. demonstrated that the overexpression of miR-21 decreases the sensitivity of advanced cervical cancer to chemoradiotherapy through the mothers against the decapentaplegic homolog 7 (SMAD7) pathway, contributing to treatment resistance. SMAD7 acts as a negative regulator of transforming growth factor β (TGF-β) activity by inhibiting the phosphorylation of SMAD2/3. This highlights miR-21 as a potential therapeutic target to enhance radiosensitivity in cervical cancer [29].

miR-21 plays a crucial role in oncogenesis by modulating several pathways. It inhibits PDCD4 and PTEN, activating the PI3K/*Akt* pathway, leading to mTORC1 activation through the inhibition of the tuberous sclerosis complex (TSC). This activation increases hypoxia-induced factor-1 alpha (HIF-1α) expression, promoting cell growth, epithelial–mesenchymal transition (EMT), and metastasis. miR-21 also upregulates TGF-β, further diminishing PTEN signaling and enhancing *Akt* activation [30]. Additionally, miR-21 reduces PDCD4’s inhibition of eukaryotic initiation factor 4A (eIF4A), further promoting cell proliferation [17]. By inhibiting GSK3β, miR-21 prevents the degradation of β-catenin, allowing for its nuclear accumulation and transcriptional activity [31]. Moreover, miR-21 downregulates FAT atypical cadherin 1 (FAT1), which stabilizes yes-associated protein (YAP)/transcriptional coactivator with PDZ-binding motif (TAZ) proteins, enhancing their activity [32]. This is further exacerbated by the downregulation of large tumor suppressor kinase 1 (LATS1), which intensifies YAP/TAZ nuclear localization and promotes radioresistance [33,34]. Human Papillomavirus type 16 (HPV16) E6 further contributes by preventing YAP degradation, amplifying its oncogenic role [35].

miR-145 is known to be a tumor suppressor and is consistently found to be low in various cancers, including cervical cancer. miR-145 mediates resistance to the cytotoxic effects of ionizing radiation. miR-145 appears to dramatically sensitize cancer cells to radiation by decreasing the efficiency of radiation-induced DNA double-strand break repair [36]. Yu et al. identified that miR-145 was downregulated in cervical cancer patients, correlating with poorer outcomes and its role as a tumor suppressor. The study highlights miR-145’s potential as a diagnostic and prognostic biomarker for cervical cancer but emphasizes the need for larger, more comprehensive research to confirm its clinical relevance [37]. The pre-radiation concentration of miR-145 in this study was expected to suppress tumor cell proliferation and produce a good radiation response, but the results showed an association with progressive disease. This suggests that the effect of tumor suppression by miR-145 may be context-dependent, requiring further investigation in larger studies.

According to recent research, miR-145-5p exhibits a dual role in colorectal cancer, acting as a tumor suppressor in the early stages by inhibiting EMT while promoting metastasis in the later stages through the activation of the *Akt* signaling pathway. This activation drives EMT-mediated anoikis resistance, allowing cancer cells to survive and spread during the advanced stages of the disease, highlighting the context-dependent nature of miR-145-5p’s function [38]. The exact mechanism by which miR-145 regulates *Akt* signaling is still unclear and requires further in vivo studies.

Another study on breast cancer found that miR-145 is associated with DNA damage response (DDR) genes, methylation patterns, and EMT. While miR-145 functions as a tumor suppressor in the early cancer stages, it can promote tumor progression in the later stages by enhancing cell proliferation and EMT [39].

**Figure 2:**
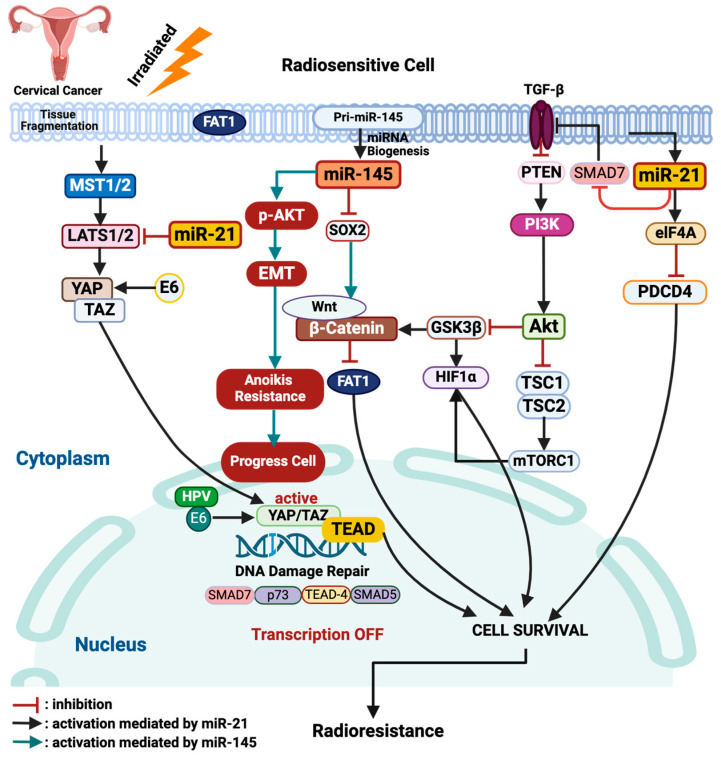
Radioresistance pathways of tumor cells mediated by miR-21 and miR-145. miR-21, microRNA-21; TGFβ, transforming growth factor β; PTEN, phosphatase and tensin homolog; PI3K, phospatidylinositol-3-kinase; *Akt*, protein kinase B; GSK3β, glycogen synthase kinase-3 beta; Wnt, wingless-related integration site; HIF-1α, hypoxia-induced factor-1α; TSC1/2, tuberous sclerosis complex 1/2; mTORC1, mammalian target of rapamycin complex 1; FAT1, FAT atypical cadherin 1; LATS1/2, large tumor suppressor kinase 1/2, eIF4a, eukaryotic initiation factor 4A; PDCD4, programmed cell death 4; E6, Early protein 6; YAP, yes-associated protein; TAZ, transcriptional coactivator with PDZ-binding motif; MST1/2, mammalian STE20-like protein kinase 1/2; SOX2, SRY-Box transcription Factor 2; TEAD-4, TEA domain transcription factor 4; SMAD5, mothers against decapentaplegic homolog 5; SMAD7, mothers against decapentaplegic homolog 7. Images created with Biorender.com.

In a study that isolated glioblastoma multiforme invasive cells, it was found that there was an overexpression of miR-145. In this study, the administration of anti-miR-145 inhibited the proliferation and metastasis of previously isolated invasive cells [40]. Naito et al. also found that the expression of miR-145 was elevated in scirrhous-type gastric cancer tissues and was closely linked to more advanced cancer stages, as well as to poorer clinical survival outcomes in patients with gastric cancer [41]. Zhang et al. demonstrated that miR-145 promotes the proliferation and metastasis of esophageal cancer cells by targeting SMAD5. Their study revealed that miR-145 overexpression contributes to tumor progression by downregulating the mothers against decapentaplegic homolog 5 (SMAD5), a gene involved in suppressing tumor growth. This indicates that miR-145 may function as an oncogene in esophageal cancer, contrary to its typical tumor-suppressive role [42]. The mechanisms of miR-21 and miR-145 described above are visually represented in Figure 2, illustrating their roles in cancer progression and treatment resistance.

miR-21 exhibits promising potential as a therapeutic target. The utilization of miR-21 as a diagnostic biomarker is extensive; however, there is a scarcity of research on its potential as a predictor of treatment response and a prognostic component. An increased expression of miR-21 can lead to resistance against chemotherapy and radiation. Hence, the potential use of anti-miR-21 therapy should be regarded as a viable option in the future [30]. Several ongoing clinical trials aim to inhibit the expression of miR-21 [43].

Despite following standardized procedures, several limitations remain, particularly the inherent complexities of molecular research, which may introduce biases in sample preparation and PCR analysis. This underscores the need for continuous refinement of protocols to ensure consistent and reliable results. Additionally, this study only measured miR-21 and miR-145 levels prior to chemoradiotherapy, missing potential changes in these biomarkers during or after treatment. Another limitation involves the follow-up method, which relied solely on telephone surveys and medical records rather than in-person medical examinations. This approach may have compromised the accuracy of the post-treatment data, as direct medical assessments would provide a more precise evaluation of patients’ statuses and treatment outcomes.

Nevertheless, this study provides valuable insights and a strong foundation for future research. The moderate predictive power of miR-21 and the limited performance of miR-145 highlight the current limitations. While combining miR-21 and miR-145 was explored as a potential way to enhance the predictive accuracy, the results showed only marginal improvements in sensitivity and specificity. Larger, more rigorous studies are needed to further clarify the clinical relevance of these miRs and to explore additional biomarkers for better predictive models for cancer diagnosis and treatment.

## 4. Materials and Methods

### 4.1. Study Design and Ethical Clearance

This analytical cross-sectional study was conducted at Cipto Mangunkusumo Hospital from July 2017 to June 2023. According to the revised 2018 International Federation of Gynecology and Obstetrics (FIGO) staging, the target population was stage IIIB and IVA cervical cancer patients who will receive definitive radiotherapy. The FIGO 2018 classification for cervical cancer is a staging system used to determine the extent of cancer spread within the body. It is essential for the purpose of guiding treatment decisions, predicting outcomes, and comparing the efficacy of treatment across various patient populations. Subject medical records were maintained under applicable medical ethical standards, and all subject-informed consent was obtained prior to research. This study was approved by the Ethics Committee of The Faculty of Medicine, Universitas Indonesia—Cipto Mangunkusumo Hospital (Ethical Clearance Certificate No: KET-245/UN2.F1/ETIK/PPM.00.02/2021).

### 4.2. Radiation Response Evaluation Criteria

The Response Evaluation Criteria in Solid Tumors (RECIST) 1.1 was used to evaluate the radiation response of cervical cancer [44]. The subjects fell into the complete response (CR) group if all target lesions disappeared and any pathological lymph nodes in the short axis were reduced to less than 10 mm. If the total diameters of the target lesions decreased by at least 30%, the subjects fell into the partial response (PR) group. Subjects were categorized in the progressive disease (PD) group if there was at least a 20% increase in the sum of the diameters of target lesions; the sum must also demonstrate an absolute increase of at least 5 mm, with a note that the appearance of one or more new lesions was also considered progression. If there was neither sufficient shrinkage to qualify for PR nor a sufficient increase to qualify for PD, it was considered a stable disease (SD).

### 4.3. Measurement of miR-21 and miR-145 Expression Levels

The expression levels were measured using real-time reverse transcriptase–polymerase chain reaction (RT-qPCR) on fresh cervical biopsy tissue. The experimental process involved the following steps: sample preparation, RNA isolation from tissue, cDNA synthesis, triplicate RT-qPCR runs, and outcome analysis. The miRNeasy Kit from Qiagen (Germantown, MD, USA) was used to isolate RNA from frozen tissue. The commercial TaqMan^®^ MicroRNA Reverse Transcription Kit from Thermo Fisher Scientific (Waltham, MA, USA) was used to reverse transcription total RNA into cDNA. Screening for miR-21 was performed using miR-21 TaqManTM MicroRNA Assays (Catalog No. #4427975, Thermo Fisher Scientific, Waltham, MA, USA) with the product name hsa-miR-21 and the mature miRNA sequence CAACACCAGUCGAUGGGCUGU. Screening for miR-145 was performed using miR-145 TaqManTM Advanced MicroRNA Assays (Catalog No. #A25576, Thermo Fisher Scientific, Waltham, MA, USA) with the product name hsa-miR-145-5p and mature miRNA sequence GUCCAGUUUUCCCAGGAAUCCCU.

The miRNA expression levels were expressed as threshold cycle (Ct) values and normalized using U6 small nuclear RNA (U6 snRNA) as the endogenous control. The ΔCt values for miR-21 and miR-145 were calculated by subtracting the Ct of U6 snRNA from the Ct of the respective miRNA (ΔCt_miR-21_ = Ct_miR-21_ − Ct_U6snRNA_ and ΔCt_miR-145_ = Ct_miR-145_ − Ct_U6snRNA_). The relative expression of each miRNA was determined using the 2^−ΔCt^ formula. These relative expression values were further normalized by dividing them by the total RNA concentration in each sample. A comparative analysis of miR-21 and miR-145 expressions across all samples was performed to evaluate the differences between patient groups.

### 4.4. Statistical Analysis

Statistical analysis was performed using SPSS Statistics version 23.0 software (IBM Corporation, Armonk, NY, USA). Categorical variables were analyzed using the chi-square test or Fisher’s exact test if the chi-square assumptions were not met. The difference in means between two groups was analyzed using the independent *T*-test for normally distributed data or the Mann–Whitney U test for non-normally distributed data. A *p*-value of less than 0.05 was considered statistically significant. The ROC curve method was used to determine the optimal cut-off values for miR-21 and miR-145 expressions.

## 5. Conclusions

This study demonstrates a moderate correlation between miR-21 expression and radiation therapy response, suggesting its potential as a biomarker for predicting radioresistance in cervical cancer. The observed correlation indicates that miR-21 could be useful in identifying patients who are less likely to respond favorably to radiation therapy, enabling more personalized treatment approaches. Conversely, miR-145 showed limited prognostic value, likely influenced by its interaction with other cancer-related genes and pathways, making it less reliable as a standalone biomarker for radioresistance. Combining miR-21 with miR-145 did not significantly enhance the predictive accuracy, reinforcing the need to explore other molecular markers for improved clinical utility.

Future studies should focus on larger, multi-center cohort studies that incorporate serial biomarker assessments of miR-21 and miR-145 expressions across various stages of cervical cancer to elucidate their roles in tumor progression and therapeutic response. Furthermore, a thorough investigation of additional genetic markers and molecular pathways that are implicated in radioresistance may facilitate the development of more comprehensive prognostic tools and optimized therapeutic strategies for cervical cancer management.

## Figures and Tables

**Table 1:** Characteristics of research subjects (*n* = 140).

Variable	Median (Q1–Q4)	*n* (%)
Age (years)	52 (44–59)	
Marital status		
No information		2 (1.4)
Not married		4 (2.9)
Married		107 (76.4)
Married > 1 time(s)		27 (19,3)
Parity	3 (2–4)	
0		8 (5.7)
1		14 (10)
2		40 (28.6)
>2		78 (55.7)
Abortion	0 (0–0)	
0		109 (77.9)
1		24 (17.1)
2		7 (5.0)

**Table 2:** The correlation between histopathology and disease staging with radiation response (*n* = 140).

Variable	*n* (%)	Radiosensitive (CR/PR) *n* (%)	Radioresistant (SD/PD) *n* (%)	*p*-Value
Histopathology				
SCC	119 (85)	47 (39.5)	72 (60.5)	0.617
AC	11 (7.9)	5 (45.5)	6 (54.5)	
Others	10 (7.1)	3 (30)	7 (70)	
FIGO Stage Classification				
IIIB	102 (72.9)	51 (50)	51 (50)	<0.001 *
IVA	38 (27.1)	4 (10.5)	34 (89.5)	

SCC: squamous cell carcinoma; AC: adenocarcinoma; CR: complete response; PR: partial response; SD: stable disease; PD: progressive disease. * significant difference in proportions, Fisher exact test.

**Table 3:** Distribution of miR-21 and miR-145 expressions by radiation response.

Variable	Staging	Radiosensitive	Radioresistance	*p*-Value *
		Median (Q1–Q4)	Median (Q1–Q4)	
miR-21 nmol/(mg/mL)	Stage IIIB	0.154 × 10^−3^ (0.00129 × 10^−3^–6.5512 × 10^−3^)	2.737 × 10^−3^ (0.180 × 10^−3^–22.424 × 10^−3^)	0.011
	Stage IVA	0.029 × 10^−3^ (0.00031 × 10^−3^–0.096 × 10^−3^)	3.582 × 10^−3^ (0.392 × 10^−3^–18.767 × 10^−3^)	0.004
	Total	0.0801 × 10^−3^ (0.0013 × 10^−3^–6.31 × 10^−3^)	2.9 ×10^−3^ (0.32 × 10^−3–^20 × 10^−3^)	<0.001
miR-145 nmol/(mg/mL)	Stage IIIB	6.3 × 10^−3^ (0.2 × 10^−3^–122.4 × 10^−3^)	25.1 × 10^−3^ (4.8 × 10^−3^–202.2 × 10^−3^)	0.081
	Stage IVA	5.0 × 10^−3^ (0.8 × 10^−3^–391.7 × 10^−3^)	57.5 × 10^−3^ (4.2 × 10^−3^–369.6 × 10^−3^)	0.407
	Total	6.4 × 10^−3^ (0.2 × 10^−3^–120 × 10^−3^)	35.2 × 10^−3^ (4.8 × 10^−3^–220 × 10^−3^)	0.029

Q1–Q4: Quartile 1–Quartile 4. Mann–Whitney Test, * significant if *p* < 0.05.

## Data Availability

The data that support the findings of this study are available from the corresponding author upon reasonable request.
